# The contribution of beta-amyloid to dementia in Lewy body diseases: a 1-year follow-up study

**DOI:** 10.1093/braincomms/fcab180

**Published:** 2021-08-19

**Authors:** Roberta Biundo, Luca Weis, Eleonora Fiorenzato, Francesca Pistonesi, Annachiara Cagnin, Alessandra Bertoldo, Mariagiulia Anglani, Diego Cecchin, Angelo Antonini

**Affiliations:** 1 Department of General Psychology, University of Padua, Padua, Italy; 2 Study Center for Neurodegeneration (CESNE), University of Padua, Padua, Italy; 3 Parkinson and Movement Disorders Unit, Department of Neuroscience, University of Padua, Padua, Italy; 4 Department of Neuroscience, University of Padua, Padua, Italy; 5 Padova Neuroscience Center, University of Padua, Padua, Italy; 6 Department of Information Engineering, University of Padua, Padua, Italy; 7 Neuroradiology Unit, Padua University Hospital, Padua, Italy; 8 Nuclear Medicine Unit, Department of Medicine—DIMED, Padua University Hospital, Padua, Italy

**Keywords:** Parkinson’s disease, dementia with Lewy bodies, amyloid, PET/MRI, cognitive dysfunction

## Abstract

Dementia in Lewy Body Diseases (Parkinson’s disease and dementia with Lewy Bodies) affects progression of disabilities, quality of life and well-being. Understanding its pathogenetic mechanisms is critical to properly implement disease-modifying strategies. It has been hypothesized that synuclein- and amyloid-pathology act synergistically aggravating cognitive decline in elderly patients but their precise contribution to dementia is debated. In this study, we aimed at exploring if presence of amyloid deposits influences clinical, cognitive and neuroanatomical correlates of mental decline in a cohort of 40 Parkinson’s disease patients with normal cognition (*n* = 5), mild cognitive impairment (*n* = 22), and dementia (*n* = 13) as well as in Dementia with Lewy Bodies (*n* = 10). Patients underwent simultaneous 3 T PET/MRI with [^18^F]-flutemetamol and were assessed with an extensive baseline motor and neuropsychological examination, which allowed level II diagnosis of mild cognitive impairment and dementia. The role of amyloid positivity on each cognitive domain, and on the rate of conversion to dementia at 1-year follow-up was explored. A Kaplan Meier and the Log Rank (Mantel–Cox) test were used to assess the pairwise differences in time-to-develop dementia in Parkinson’s disease patients with and without significant amyloidosis. Furthermore, the presence of an Alzheimer’s dementia-like morphological pattern was evaluated using visual and automated assessment of T_1_-weighted and T_2_-weighted MRI images. We observed similar percentage of amyloid deposits in Parkinson’s disease dementia and dementia with Lewy Bodies cohorts (50% in each group) with an overall prevalence of 34% of significant amyloid depositions in Lewy Body Diseases. PET amyloid positivity was associated with worse global cognition (Montreal Cognitive Assessment and Mini Mental State Examination), executive and language difficulties. At 12-month follow-up, amyloid positive Parkinson’s disease patients were more likely to have become demented than those without amyloidosis. Moreover, there was no difference in the presence of an Alzheimer’s disease-like atrophy pattern and in vascular load (at Fazekas scale) between Lewy Body Diseases with and without significant amyloid deposits. Our findings suggest that in Lewy Body Diseases, amyloid deposition enhances cognitive deficits, particularly attention-executive and language dysfunctions. However, the large number of patients without significant amyloid deposits among our cognitively impaired patients indicates that synuclein pathology itself plays a critical role in the development of dementia in Lewy Body Diseases.

## Teaser

Biundo et al. used PET/3TMRI to study amyloidosis influence on cognition and conversion to dementia in Lewy Body disorders. A similar percentage of patients were amyloid-β negative, but general cognition was more severe and conversion to dementia was faster in amyloid-β positive. Amyloid-β contributes to cognitive decline in Lewy Body disorders.

## Graphical Abstract

**Graphical Abstract:**
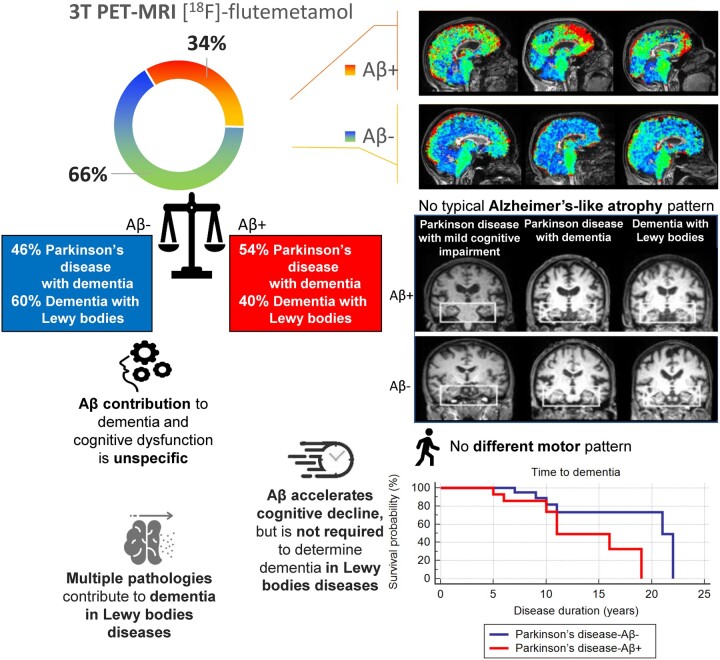

## Introduction

Lewy Body Diseases, such as Parkinson’s disease and Dementia with Lewy bodies, manifest with different phenotypes and a combination of motor and non-motor symptoms. As research is increasingly focussed on disease modifying strategies, identifying symptoms that most contribute to disease progression has become a key objective in translational research in both conditions.^1–3^

Cognitive decline and dementia are established disease milestones in Parkinson’s disease as they negatively impact on progression of disability,^2^^,^^4^ quality of life^4–6^ and life expectancy.^7^

Parkinson’s disease with mild cognitive impairment (PD-MCI) and particularly deficits in semantic and visuospatial skills have been associated with faster cognitive decline and conversion to dementia,^8–11^ but the significant variability in the PD-MCI profile makes prediction very challenging in individual patients.^12^

The underlying pathology is also heterogeneous with amyloid plaques, tau neurofibrillary tangles (Alzheimer’s hallmarks), cortical Lewy bodies^13^^,^^14^ reported with variable frequency in demented patients.

Neuropathology studies suggest that in Parkinson’s disease amyloidosis is associated with worse cognitive status and shorter motor to dementia interval, similarly to dementia with Lewy Bodies.^15^ According to the National Institute of Aging and Alzheimer’s Association research framework, brain amyloid deposition only determines whether or not an individual is in the Alzheimer’s continuum, and by itself is not sufficient for the diagnosis of Alzheimer’s disease as well as the presence of neurodegenerative biomarkers and cognitive symptoms.^16–19^ Moreover, 30–40% of cognitively unimpaired elderly persons have Alzheimer’s disease neuropathologic changes at autopsy,^20^ and a similar proportion has abnormal amyloid biomarkers.^21^ Based on these findings, a synergism between synuclein and Alzheimer’s disease-type pathologies [primarily amyloid deposits (Aβ) pathology] to dementia-risk in Lewy Body Diseases is conceivable.^22^^,^^23^ Furthermore, amyloidosis increases with ageing, highlighting the importance to investigate patients in a specific age range.^24^

However, most published studies on Lewy Body Diseases include relatively small cohorts with frequently incomplete cognitive characterization, and heterogeneous patients’ age, making difficult to draw firm conclusions about the role of amyloid in Lewy Body Diseases.^25–29^

In this study, we assessed amyloid deposition by [^18^F]flutemetamol PET and investigated if this distinguishes clinical/cognitive phenotypes in patients with the whole spectrum of Parkinson’s disease and dementia with Lewy Bodies (DLB) [PD cognitively intact (PD-NC), PD-MCI, Parkinson’s with dementia (PDD) and DLB] and within a similar age range. Since imaging was performed using a simultaneous 3T PET/MRI scanner, we evaluated in the same patient, structural and molecular changes and if this is associated with prevalence of dementia at baseline and prospectively at 12-month follow-up.

## Material and methods

### Study population

**Figure 1:**
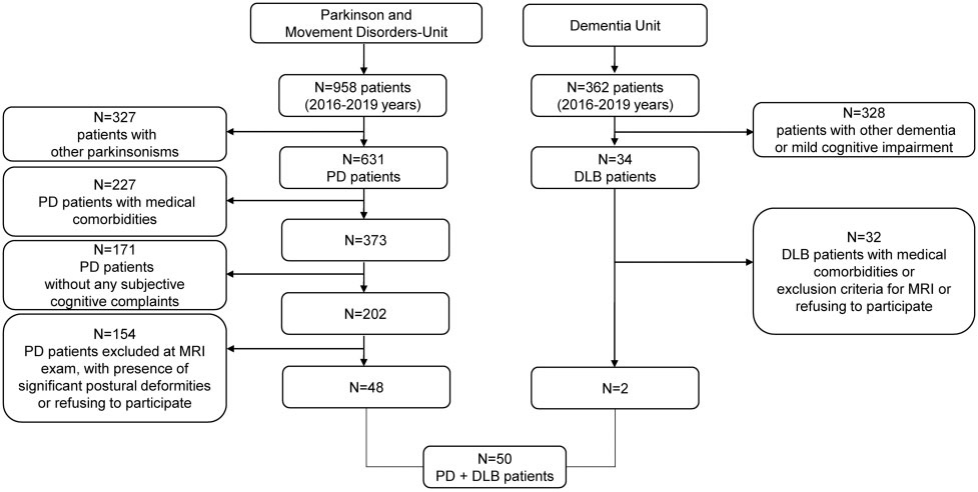
**Flowchart of the studied population**.

A total of 50 patients [10 DLB, 13 PDD, and 27 non-demented Parkinson’s disease (22 PD-MCI and 5 PD-NC)] were included in the study from 2016 to 2019. Patients were recruited consecutively at the Parkinson’s Disease and Movement Disorders Unit and Dementia Unit of Neurology Clinic in Padua and underwent a comprehensive clinical and neuropsychological evaluation. We considered only PD and DLB patients within an age range between 55 and 85 years, encompassing the overall spectrum of cognitive status in Lewy Body Diseases, with subjective complains and/or early dementia (based on altered activities of daily living). From a total of 1000 patients evaluated at first visit, 50 subjects were included in the study (please see Fig. 1 with the flowchart detailing the recruitment phase).

All Parkinson’s disease patients fulfilled the Queen Square Brain Bank criteria for the diagnosis of probable Parkinson’s disease^30^; the clinical diagnosis of probable DLB was based on published consensus criteria.^31^ All DLB patients had parkinsonism and presented dementia either before or within 1 year from the onset of motor manifestations. The clinical diagnoses for both PD and DLB were confirmed by presence of reduced DaTscan SPECT binding in the striatum. Exclusion criteria were the treatment with deep brain stimulation, atypical Parkinsonian disorders, severe psychiatric or neurological comorbidity, and clinically relevant cerebrovascular disease on MRI. The study was approved by the Ethic Committee of the University of Padua (4340/AO/17). All patients gave written informed consent according to the Declaration of Helsinki.

### Clinical and neuropsychological examination

Demographic, clinical characteristics and presence of comorbid diseases were collected by neurologists with experience in movement disorders.

Disease severity was assessed using the motor section of the Movement Disorder Society Unified Parkinson's Disease Rating Scale (MDS-UPDRS-III)^32^ and Hoehn–Yahr scale. Motor phenotypes were determined as either tremor dominant phenotype or postural instability and gait disturbance phenotype following the classification algorithm proposed by Jankovic et al.^33^ Following the original classification methods, the ratio of the mean MDS-UPDRS-III tremor scores (items: 15–18) to the mean MDS-UPDRS gait disturbance scores (items: 9–12) was used to define tremor phenotype (ratio ≥ 1.5), gait disturbance phenotype (ratio ≤ 1) or indeterminate phenotype (ratios >1.0 and <1.5). Finally, the sum of items 3.3a–3.3e, and sum of items 3.7a–3.8b and 3.14 were considered for rigidity and for bradykinesia. The score for each motor symptom was calculated by summing up the scores of corresponding items in MDS-UPDRS-III (see Supplementary S1).

Levodopa and dopamine agonist equivalent daily doses were calculated.^34^ Seven out of 23 demented patients (5/13 PDD and 2/10 DLB) were on acetylcholinesterase inhibitors and one on memantine.

Patients underwent an extensive neuropsychological examination to diagnose mild cognitive impairment and dementia according to the Movement Disorder Society Level II criteria^35^^,^^36^ (for further details on cognitive tests adopted, see Fiorenzato et al.^37^) In respect to the extensive neuropsychological assessment, the Trail making Test-B was considered ‘not feasible’ as more than the 20% of patients in both groups were not able to either initiate or complete the task and for this reason was excluded from the analyses. Age at disease onset was defined as the age at which the patient noticed the first motor symptom (or cognitive symptoms in DLB subjects) indicative of Parkinson’s disease. The expert neuropsychologist (who assessed the II level mild cognitive impairment patient at baseline), blinded to motor score and PET results, made a diagnosis of cognitive stable versus converted to dementia profile at 1-year follow-up interval. Dementia diagnosis was made whether caregivers reported significant changes in patient’s activities of daily living (altered ADLs).^38^

The ADLs scale, developed by Katz,^39^ is the most frequently used measure of older adults’ basic functional status. This scale can be self-administered or assessed by caregiver or a healthcare worker. The inability to perform ADLs results in the dependence of other individuals and/or mechanical devices. The basic ADLs are those skills required to manage one’s basic physical needs, including personal hygiene or grooming, dressing, toileting, transferring or ambulating, and eating. The total score for ADLs is 0–6. When this screen is used over time, it serves to document a person’s functional improvement or deterioration.^40^

Well-being and quality of life were assessed using the Parkinson’s Disease Questionnaire (PDQ-8).^41^ The presence of depression, anxiety and apathy was assessed using the Beck Depression Inventory-II (BDI-II), State-Trait Anxiety Inventory forms (STAI Y-1 to assess state anxiety, STAI Y-2 to assess trait anxiety) and Starkstein Apathy Scale (AS), respectively.^42^

Patients were evaluated in ‘on’ medication state. The cognitive tests were administered by trained neuropsychologists, in the morning, on two separate occasions within 3–5 days. All clinical assessments were performed blind to PET results.

### PET/MRI acquisition

Lewy Body Diseases participants in accordance with the amyloid imaging procedure guideline^43^ received an intravenous injection of about 185 MBq [^18^F]flutemetamol (performed manually over 10 s and flushed with 30 ml of saline over about 15 ± 5 s) directly in an integrated 3T PET/MRI system (Biograph mMR; Siemens, Erlangen, Germany). Images were acquired between 0–10 and 90–110 min after injection according to Cecchin et al.^44^ PET data were reconstructed into a 256 × 256 matrix (voxel 2.32 × 2.32 × 2.03) using the built-in 3D Ordinary Poisson-Ordered Subset Expectation Maximization algorithm with eight iterations, 21 subsets and a 3-mm Gaussian post-filtering. Standard correction of decay, scatter, dead time and attenuation was performed. An ultrashort echo time sequence was used for attenuation correction. Anatomical volumetric data via T_1_-weighted-3D magnetization-prepared rapid acquisition gradient echo sequence (TR 1.900 ms, TE 2.53, slice thickness 1 mm, matrix 256 × 256, FOV 250 mm) were simultaneously acquired. Additionally, a 1 mm-isotropic T_2_-weighted-3D, and two-dimensional susceptibility-weighted imaging were acquired for clinical evaluation, excluding secondary parkinsonisms, the presence of vascular brain damage and allowing visual rating scales assessment.

### Classification of PET amyloid images

Binary visual classification of [^18^F]flutemetamol scans as positive or negative is accurate and reliable for detection of cases with histology defined plaques.^45^ An expert nuclear medicine physician (DC, with both in-person and e-training), blinded to cognitive status and diagnosis, rated each scan as amyloid-positive (Aβ+) or negative (Aβ−). This judgement was based on the assessment of [^18^F]flutemetamol uptake in grey versus white matter in frontal lobes, anterior cingulate, posterior cingulate, precuneus, temporo-parietal, including insula, lateral temporal lobes and striatal region (in accordance with [^18^F]-Flutemetamol product information sheet: https://www.ema.europa.eu/en/medicines/human/EPAR/vizamyl).

### Visual Alzheimer's disease-like pattern assessment

Two trained observers (L.W. and A.A.) independently visually rated each participant T_1_-weighted and T_2_-weighted MRI images blinded to age, sex or diagnosis and rated the Scheltens’ scale of medial temporal atrophy (MTA),^46^ the Pasquier’s Global Cortical Atrophy scale and the frontal subscale (GCA and GCA-F, respectively),^47^ the Koedam’s scale of Posterior Atrophy (PA) and the Fazekas’s scale.^48^^,^^49^ In order to increase the raters’ accuracy,^50^ all brains MRI data were rigidly realigned to the Montreal Neurologic Institute template using FMRIB’s Linear Image Registration Tool (FLIRT-FSLv6.0, https://fsl.fmrib.ox.ac.uk/fsl/fslwiki).^51^^,^^52^ This rigid transform was computed with six degrees of freedom (i.e. rotation and translation only) and was used to align each brain automatically to the anterior commissure—posterior commissure line and conform all images to the same voxel size (1 mm^3^ × 1 mm^3^ × 1 mm^3^) and input dimension (182 × 218 × 182). Cut-offs for the visual rating scales have previously been published.^53^ Normal versus abnormal cut-off points were determined for each individual in the four visual rating scales. An MTA score ≥1.5 was considered abnormal in the age-group 65–74, ≥2 for the age-group 75–84 and ≥2.5 for the age-group 85–94. For PA and GCA and GCA-F, a score ≥1 was always considered abnormal irrespective of age (age-correction did not improve diagnostic accuracy in cut-offs derivation). For Fazekas’s scale, a score ≥1 was considered indicative of at least mild vascular abnormalities.^48^ The combination of MTA, PA and GCA-F and Fazekas score have previously been described in relation to Alzheimer's disease.^53^ Namely, we identified (i) *the Alzheimer's disease-like pattern* if a patient presented either the limbic-predominant pattern (defined as an abnormal MTA, and a normal PA and GCA-F) or the typical Alzheimer's disease pattern (defined as an abnormal MTA in conjunction with either an abnormal PA or GCA-F or an abnormal MTA in conjunction with both an abnormal PA and GCA-F) or the hippocampal-sparing pattern (defined as a normal MTA and either an abnormal PA or GCA-F, or both an abnormal PA and GCA-F); and (ii) the *Alzheimer's disease-like pattern plus Fazekas.*^54^

### Automated Alzheimer's disease-like pattern assessment

The Brain Anatomical Analysis using Diffeomorphic deformation (BAAD 4.31-http://www.shiga-med.ac.jp/~hqbioph/BAAD/Welcome_to_BAAD.html) Statistical Parametric Mapping tool (SPM12, https://www.fil.ion.ucl.ac.uk/spm/software/spm12/) was used to calculate the Alzheimer's disease-score from T_1_-weighted-3D in order to predict the presence of an early-Alzheimer's disease pattern. This tool includes a Computational Anatomy Toolbox (CAT12)-based (http://www.neuro.uni-jena.de/cat/), T_1_-weighted-3D diffeomorphic segmentation after inhomogeneity correction, T_1_-weighted-3D quality check assessment and Total Intracranial Volume (TIV) estimation. T_2_-weighted-3D was included in the multimodal segmentation for correcting brain atrophy estimation from the presence of white matter lesions. BAAD’s Regions of Interest extraction integrated the Wfu_Pickatlas (http://fmri.wfubmc.edu/software/PickAtlas) and MarsBaR (http://marsbar.sourceforge.net) SPM12 toolboxes. Alzheimer's disease score is a score ranging from zero to one. With a score higher than 0.6, the patient is highly suspected as being in the Alzheimer's disease continuum.^55^ This score comes from multiple sampling points of brain volume of interest (VOIs) and judged by support-vector machine (SVM) model. The SVM model provided in BAAD was previously trained, including 700 patients using Alzheimer Disease Neuroimaging Initiative (ADNI, http://adni.loni.usc.edu/) data of North America and validated by Australian Imaging, Biomarker & Lifestyle Flagship Study of Ageing (AIBL, https://aibl.csiro.au/) or Japanese ADNI (http://www.j-adni.org/) data. Sequential minimal optimization was used for solving the minimization SVM problem and removing outliers with the gradient difference as convergence criteria. The Alzheimer's disease-score provided by BAAD was estimated based on 10 VOIs of the Automated Anatomical Labeling atlas^56^: right BA28, left amygdala, left angular gyrus, left frontal inferior operculum, left superior medial frontal + right superior medial frontal, left middle occipital, left superior parietal, left Rolandic operculum and left middle temporal pole. Age, TIV and sex were included in the predictive model based on age and sex ranked healthy subject normative data. For explorative purpose, BAADs tool provide at subject level a voxel-wise non-parametric statistical map of grey matter and white matter alterations, comparing each participants brain MRI normalized and segmented image to the age- and sex-matched normative data with the SnPM12 tool (http://warwick.ac.uk/snpm).

### Statistical analysis

An a priori power analysis was conducted using G*Power3.1.9.4^57^ to test the difference between Montreal Cognitive Assessment (MoCA) score means in two independent groups using a two-tailed Wilcoxon–Mann–Whitney test. The asymptotic relative efficiency (A.R.E. method) was used for effect size estimation. A large effect size (*d* = 1.027) and allocation ratio N1/N2 = 0.5 were calculated based on mean (SD) MoCA scores and Aβ+ frequency reported in the Parkinson’s Progressive Markers Initiative large Parkinson’s disease cohort study.^58^ An alpha probability error of 0.05 was considered for sample estimation. Results showed that a total sample of 50 participants with 17 Lewy Body disorders with significant Aβ (LBDs-Aβ+ subgroup) and 33 Lewy Body disorders without significant Aβ (LBDs-Aβ− subgroup) was required to achieve a power of 0.91.

Pairwise differences in characteristics (clinical, motor and cognitive) between the two Lewy Body disorders subgroups (LBDs-Aβ+ versus LBDs-Aβ−) were assessed with Mann–Whitney U-test or Fisher’s exact test as appropriate. In order to explore the role of Aβ on each cognitive domain and cognitive test performance, we converted each cognitive raw score to *Z*-score using Italian normative data. Then, we calculated the *Z*-compound as the mean *Z*-score among tests of each cognitive domain and considered −1.5 SD as pathologic performance cut-off. Pearson’s Chi-square statistic was used to compare percentage of failures in LBDs-Aβ+ versus LBDs-Aβ−. Spearman’s chi-squared test was run to explore whether Aβ+ is associated with an MRI AD-like pattern in PD-MCI and Lewy Body dementia subgroups (PDD and DLB). Rate of conversion at 1-year FU was calculated and difference in rate of conversion in Parkinson’s disease subgroups (PD-Aβ+ versus PD-Aβ−) were evaluated using the McNemar test. Finally, a Kaplan–Meier and the log-rank (Mantel–Cox) test were used to assess the pairwise differences in time-to-develop dementia from disease onset in PD-Aβ+ versus PD-Aβ− subgroups. Moreover, the relative risk of hazard occurring at any given time in PD-Aβ+ to develop dementia was calculated. Statistical analyses were run using IBM-SPSS 25 (IBM SPSS Inc., Chicago, IL, USA) and python 3.8 seaborn libraries for violin plot distribution density.

### Data availability

All data are available upon reasonable request.

## Results

### Patients’ characteristics at baseline

Thirty-three out of 50 Lewy Body Disorders patients presented with Aβ− status (5 PD-NC, 16 PD-MCI, 6 PDD and 6 DLB) and 17 with Aβ+ status (6 PD-MCI, 7 PDD and 4 DLB). Because the PDD and DLB groups did not differ in terms of cognitive and neuropsychiatric functioning or percentage of positive [^18^F]flutemetamol uptake, we pooled them into a single Lewy body type dementia (DEM) group (13 DEM Aβ− versus 10 DEM Aβ+) for the remaining analyses (see Supplementary Table 1). The overall Lewy Body disorders subgroups (5 PD-NC, 16 PD-MCI and 13 DEM Aβ− versus 6 PD-MCI and 10 DEM Aβ+) did not significantly differ in any general clinical characteristic except for Mini Mental State Examination (MMSE) and MoCA total score. Moreover, they were indistinguishable when detailed motor symptoms were compared (see Table 1 and Supplementary Fig. 1 for more details).

**Table 1:** Demographical, motor and clinical features (Mean, SD) in LBDs-Aβ+ versus LBDs-Aβ−

	LBDs		Mann–Whitney U-test
	LBDs-Aβ− (*n* = 33)	LBDs-Aβ + (*n* = 17)	*P*-value
Demographical			
Age (years)	70.12 (7.28)	72.94 (4.08)	0.1189
Sex (Male)	69%	76%	0.7455
Education (years)	10.46 (5.30)	9.75 (5.25)	0.5525
Clinical characteristics			
Age at symptoms’ onset (years)	60.49 (8.89)	64.88 (5.878)	0.0527
Disease duration < 5 years (%)	18%	24%	0.7172
Disease duration (years)	10.00 (6.39)	8.47 (5.01)	0.4790
LEDD (mg tot/die)	752.78 (458.17)	701.50 (389.85)	0.7606
DAED (mg tot/die)	89.03 (105.42)	67.29 (99.20)	0.5372
DA (%)	56	53	0.9999
RBD (%)	56	56	1.000
Hallucination (%)	42	64	0.2321
Subjective complain (%)	61	77	0.3510
Motor characteristics			
H&Y > 3 (%)	42	47	0.7689
MDS-UPDRS total score	67.17 (38.43)	53.0 (28.62)	0.5156
MDS-UPDRS-III	33.55 (19.44)	38.94 (23.63)	0.4792
PIGD/TD/indeterminate phenotype (%)	91/0/9	100/0/0	0.5133
MDS-UPDRS-III-Bradykinesia (%)	70	82	0.4992
MDS-UPDRS-IV-Dyskinesias (%)	19	8	0.6426
MDS-UPDRS-IV-Fluctuation (%)	54	50	0.9999
Functional activities			
ADL	4.31 (1.75)	4.00 (1.80)	0.4958
IADL	3.59 (1.95)	2.77 (1.99)	0.1244
PD-CFRS	9.03 (7.50)	11.68 (6.97)	0.2263
Global cognitive status			
MMSE (corrected score)	22.85 (3.78)	20.17 (4.43)	0.0366*
MoCA (corrected score)	18.48 (4.70)	14.97 (3.78)	0.0183*

DAED, dopamine agonist equivalent daily dose; DA (%), percentage of patients in dopamine agonist therapy; H&Y, Hoehn and Yahr scale; IADL, Instrumental ADL; LEDD, levodopa and dopamine agonist equivalent daily doses; PIGD phenotype (%), percentage of postural instability and gait disturbance; RBD (%), percentage of rapid eye movement sleep Behaviour Disorder; TD phenotype (%), percentage of tremor dominant disturbance; *P < 0.05.

**Figure 2:**
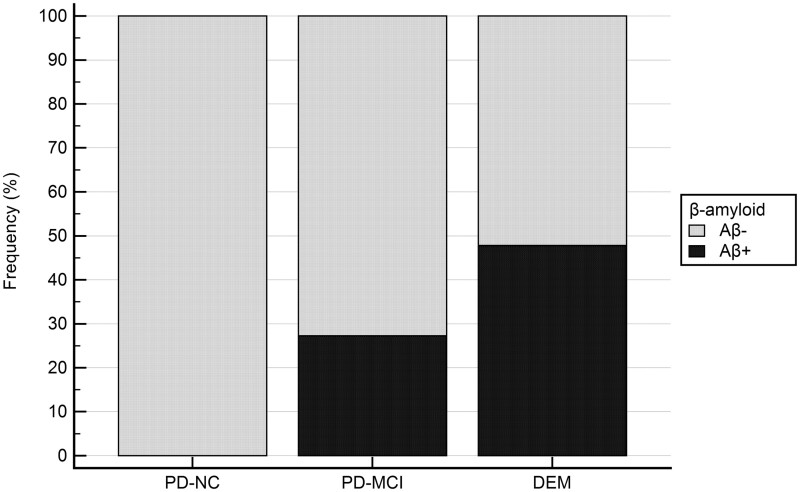
**Percentage of Aβ in LBDs patients across cognitive states.** Histogram plot of percentage of Aβ+ (black) in LBDs patients. Namely, 0% of Aβ+ in PD-NC, 27.3% Aβ+ in PD MCI and 47.8% Aβ+ in DEM. Chi-square analyses comparing presence/absence of AB is not significant.

LBDs-Aβ+ patients showed worse global cognition at MoCA (*P* = 0.0183) and at MMSE (*P* = 0.0366) scales. Looking at specific test abilities, LBDs-Aβ+ versus LBDs-Aβ− showed worse performance in the executive functions as measured by the Wechsler Adult Intelligence Scale Similarities subtest (*P* = 0.0486) and by the Stroop Error test (*P* = 0.0343) and in the overall language domain (*P* = 0.0177), whose *Z*-compound score is driven by category fluency plus naming task performance (see Table 2). Finally, analyses of cognitive statuses revealed increased Aβ+ percentage according to cognitive severity (0% versus 27.3% versus 47.8% in PD-NC, PD-MCI and DEM, respectively), although Chi-squared test was not significant (see Fig. 2).

**Table 2:** Comparison (percentage) of LBDs subgroups (Aβ− versus Aβ+) performing below −1.5 *Z*-score in each cognitive test

	LBDs		Fisher’s exact test
Cognitive domains	LBDs-Aβ− (*n* = 33)	LBDs-Aβ+ (*n* = 17)	*P-*value
Attention/working memory			
TMT-A	43.7%	64.7%	0.2320
DSST	28.1%	47.1%	0.2190
*Z*-compound	69.7%	88.2%	0.1811
Executive			
Stroop/color task-Time	43.7%	41.2%	0.9999
Stroop/color task-Error	50.0%	82.4%	0.0343^*^
Phonemic Fluency	25.0%	17.6%	0.7250
Similarities	9.4%	35.3%	0.0486^*^
CDT	25.0%	41.2%	0.3314
*Z*-compound	60.6%	47.1%	0.3857
Memory			
Prose Memory Test	65.6%	82.4%	0.3228
Prose Memory Test delayed	62.5%	76.5%	0.3601
ROCF delayed	48.4%	70.6%	0.2237
WPAT	48.4%	70.6%	0.2237
*Z*-compound	36.4%	58.8%	0.1473
Language			
Naming Task	38.7%	52.9%	0.3775
Category Fluency	37.5%	41.2%	0.9999
*Z*-compound	39.4%	76.5%	0.0177^*^
Visuospatial			
VOSP	56.2%	68.7%	0.5341
Benton	62.5%	82.4%	0.2016
ROCF copy	71.0%	88.2%	0.2839
*Z*-compound	45.5%	41.2%	0.9999
Apraxia			
Apraxia	24.1%	41.2%	0.3215

CDT, Clock drawing test; DSST, Digit Sequencing Symbol test; ROCF copy, Rey–Osterrieth complex figure test (immediate copy); ROCF delayed, Rey–Osterrieth complex figure test (delayed copy); TMT-A, Trail making test part A; VOSP, Visual Object and Space Perception Battery; WPAT, Word paired associated test. *P < 0.05.

From a behavioural point of view, we did not find any significant differences between the two subgroups in depressive symptoms [BDI-II mean (SD): 8.46 (6.50) for Aβ− versus 9.67 (5.45) for Aβ+], anxiety [STAI Y-1 mean (SD): 36.96 (8.46) for Aβ− versus 38.08 (6.16) for Aβ+; STAI Y-2 mean (SD): 42.50 (10.21) for Aβ− versus 40.17 (10.36) for Aβ+], apathy [AS mean (SD): 17.71 (7.16) for Aβ− versus 18.27 (5.49) for Aβ+] and well-being [PDQ-8 mean (SD): 9.16 (5.64) for Aβ− versus 10.60 (7.26) for Aβ+].

### Longitudinal analyses

At 1-year follow-up [mean: 14 months (8.00)], the 22 PD-MCI conversion rates to dementia were significantly greater in PD-Aβ+ (*N* 6) versus PD-Aβ− (*N* 16) (*P* < 0.0036) [0% remained stable and 100% converted (*N* 6) versus 81.25% remained stable (*N* 13) versus 18.75% converted (*N* 3), respectively].

**Figure 3:**
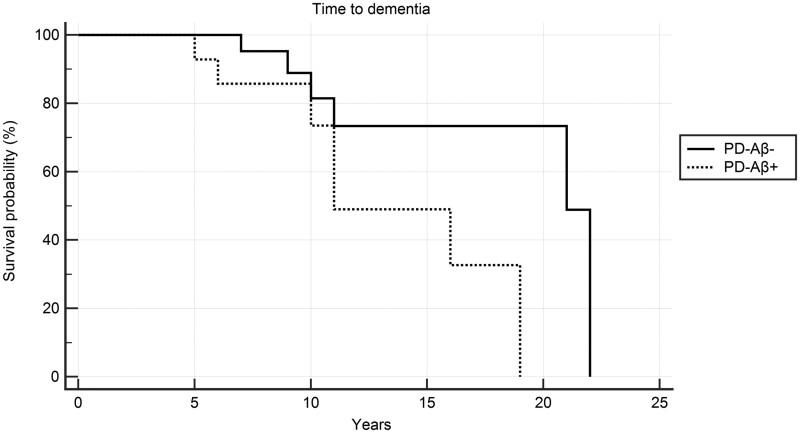
**Impact of Aβ on time to develop dementia in PD patients.** Plot of Kaplan–Meier limit estimates of time to develop dementia in PD-Aβ+ versus PD-Aβ−. Logrank test *P* = 0.0359. Aβ+ increased Hazard ratios with 95% Confidence Interval of developing dementia = 2.83 (0.86–9.36).

Log-rank test of the 14 PD-Aβ+ and 26 PD-Aβ− showed that the presence of Aβ anticipated the time-to-dementia (*P* < 0.0359). Moreover, PD-Aβ+ showed a Hazard ratio of 2.83 (CI 0.86–9.36) to develop dementia as compared to PD-Aβ− (see Kaplan–Meier curve in Fig. 3).

### MRI Alzheimer's disease-like pattern

**Figure 4:**
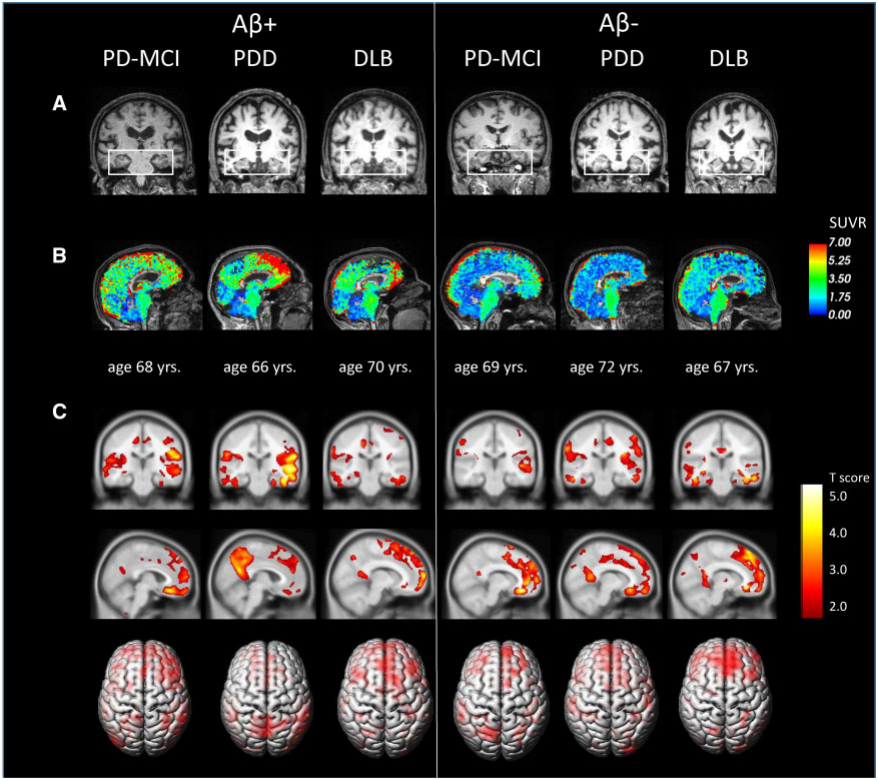
**Representative images of LBDs patients according to Aβ status.** (**A**) Visual assessments of Medial temporal lobe Index (MTA) in PD and DLB patients with/without Aβ, showing absence of mild to moderate MTA atrophy. Coronal T1w-3D views. (**B**) PET-MRI [^18^F]Flutemetamol SUVR maps obtained with cortical cerebellum as reference after three compartment Müller-Gärtner partial volume correction with PETsurfer. SUVR maps are overlaid onto each participant’s native T_1_-weighted 3D, showing significant Aβ deposits in frontal–striatal regions, anterior and posterior cingulate areas and precuneus. (**C**) Statistical non-parametric maps of significant voxel-based grey matter atrophies in frontal–striatal regions, anterior and posterior cingulate, precuneus and middle temporal areas in LBDs. Statistical maps are overlaid onto the standard MRI template after normalization to the MNI space using CAT12 tool pipeline.

Forty-seven out of 50 Lewy Body Disorders patients {31 with Aβ− status [5 PD-NC, 15 PD-MCI, 11 DEM (5 PDD and 6 DLB)]} and 16 with Aβ+ status [6 PD-MCI, 10 DEM (7 PDD and 3 DLB)]} were included in the analysis. Three patients were excluded due to poor quality MRI resolution (presence of movement’s artefacts). We used a visual as well as automated assessment of T_1_-weighted and T_2_-weighted MRI images to identify presence of an Alzheimer's disease-like pattern. Overall, <40% of the sample showed a visually rated Alzheimer's disease-like pattern, and ∼50% of PD-MCI and DEM showed an early Alzheimer's disease-like automated score predictive of dementia. The three calculated indexes (the Alzheimer's disease-like pattern, the Alzheimer's disease-like patter plus Fazekas and the early Alzheimer's disease-like automated) were equally represented in each LBDs-Aβ subgroup (see Table 3). For a detailed summary of percentages distribution of GCA, CGA-F, MTA (left and right), PA (left and right) and Fazekas score in PD-MCI and DEM subgroups (see Supplementary Table 2). Please see Fig. 4 for representative T_1_-weighted 3D and PET amyloid images showing a) absence of MTA atrophy, b) FMM uptake in selected LBDs cases c) significant atrophy in fronto-striatal, anterior and posterior cingulate, precuneus and middle temporal regions compared to normative data.

**Table 3:** Percentage (%) of AD-like pattern in LBDs-Aβ subgroups

	LBDs		Fisher's exact test
	LBDs-Aβ− (*n* = 31)	LBDs-Aβ+ (*n* = 16)	*P*-value
AD-like VR pattern (%)	32.3	37.3	0.7539
AD-like VR pattern plus Fazekas (%)	6.5	0	0.5411
Early AD-like automated pattern (%)	33.3	50.0	0.3470

AD-like VR pattern: patients with an AD-like pattern according to the presence of moderate to severe combination of MRI atrophy at visual rating scales (Global cortical atrophy-Frontal subscore, Middle temporal atrophy, posterior Atrophy-Koedam-score); AD-Like VR pattern plus Fazekas: patient with an AD pattern based on visual rating scale and a moderate to severe subcortical vascular alteration; Early AD-like automated pattern: patients with an early AD-like pattern based on an automated AD-score index using a voxel based-MRI segmentation approach.

## Discussion

We investigated the contribution of amyloid deposition on clinical and cognitive performance and the interplay with brain atrophy in a cohort of cognitively well-characterized age-matched patients with Lewy Body Disorders using simultaneous PET/MRI imaging.

In our cohort, the overall pooled prevalence of positive cerebral PET amyloid uptake was 34% of Lewy Body Disorders patients. Interestingly, there was a similar proportion of positive and negative amyloid scans among demented participants (54% of PDD and 40% of DLB). PET amyloid positivity was associated with worse cognitive status, increased global cognitive impairment and alterations in attentive/executive and language functioning.

These findings corroborate previous evidence showing similar Aβ prevalence in Parkinson’s disease and elderly controls and a significant positive relationship between cortical beta-amyloid deposition and general cognitive impairment in Lewy Body Disorders.^21^^,^^58–60^ Moreover, our PD-Aβ+ patients showed faster turning to dementia than PD-Aβ−, supporting the concept that Aβ pathology is associated with more rapid cognitive decline.^61–65^ In addition, PET amyloid positivity was associated with a trend of worse cognitive status (0% versus 27.3% versus 47.8% in PD-NC, PD-MCI and DEM, respectively).

We also found negative cerebral amyloid uptake in both demented and PD-MCI patients who after 1 year turned to dementia. Indeed, a systematic review of more than 2000 autopsy brains in PDD, AD and DLB showed presence of limbic and neocortical Lewy body pathology in all PDD cases, while moderate or severe Aβ pathology was observed in only half of the cases and tau in only one-third.^65^ Moreover, high concentration of cortical Lewy bodies pathology best predicts dementia in DLB, and this correlates with clinical DLB symptoms.^13^ These findings support previous clinicopathological studies suggesting that multiple underlying pathologies contribute to dementia in PD, including Lewy Body pathology.^66–69^ Unfortunately, given the lack of dedicated imaging tracers we cannot document cerebral distribution of synuclein pathology and assess if it is responsible of the variability in dementia rates, we observed in our patients with Lewy Body Disorders.

Neuropsychological data showed that amyloidosis in Lewy Body Disorders patients worsens general cognition (measured by means of MoCA and MMSE scales), attentive–executive abilities (in particular, verbal reasoning and abstraction, cognitive flexibility, processing speed and response inhibition behaviour) as measured by Similarities and Stroop Error tasks; and language functioning (specifically verbal recall and picture naming) as assessed by category fluency and naming tasks.

These findings are also in line with our previous studies showing that both MoCA and MMSE are feasible scales to assess cognitive alterations in presence of frank dementia.^12^^,^^70^ However, in Lewy Body Disorders, MoCA is more sensitive and clinically useful cognitive screening instrument^9^^,^^71^ and correlates well with CSF Aβ 1–42 levels.^58^^,^^72^ Moreover, attentive/executive dysfunctions have been already observed in Aβ-positive elderlies without dementia,^73^ and in Aβ+ early-stage Parkinson’s disease patients with increased cortical and subcortical amyloid depositions.^58^

In our study, we also found significantly altered language abilities in LBDs-Aβ+ specifically by using tasks requiring access to semantic world knowledge. Evidence identified in the semantic recall dysfunctions a strong risk factor of the development of Parkinson’s disease related dementia, with these impairments reflecting a posterior temporal spread of Parkinson’s disease pathology.^9^^,^^74^ Moreover, it has been recently observed that markers of Alzheimer's disease-type co-pathology are implicated in impaired language performance in Lewy Body Disorders^75^ and that alterations in semantic fluency and naming tasks in these patients are associated with both Lewy body and Alzheimer's disease neuropathology in limbic/temporal areas.^29^^,^^76–78^

Interestingly, there was no difference in proportion of patients presenting Alzheimer's disease-like atrophy pattern at MRI in LBDs-Aβ+ versus LBDs-Aβ− patients with and without dementia. Cognitive performance including long-term memory was also similar in line with the concept that amyloid plaques are not the primary driver of dementia in LBDs, but they contribute in conjunction with alpha-synuclein and possibly hyper-phosphorylated tau deposition. Moreover, the lack of significant hippocampal atrophy and memory dysfunctions in our LBDs-Aβ+ patients, well supports data from a recent neuropathological study showing that tau pathology but not Aβ42 levels correlates with hippocampal volume and general cognitive status in non-demented Parkinson’s disease.^79^

Our study also provides some ground in the debate of PDD/DLB single versus different neuropathological aetiology. The Diagnostic Manual of Mental Disorders-fifth edition lists these two neurocognitive disorders as two separate entities. However, they share similar morphological hallmarks (cortical–subcortical alpha-synuclein, Lewy-body plus beta-amyloid and, to a less extent, plus tau pathologies),^80^ but different clinical onset and cognitive severity profile^70^ indicating some dissimilarities. By adopting the common convention of the 1-year rule, the major conclusion is that DLB shows a more severe neuropsychiatric phenotype than PDD.^70^^,^^81^ Neuropathological findings do not always support the arbitrary temporal distinction, with some studies observing substantial overlap of PDD and DLB phenotypes in many clinical and neuropathological investigations,^82–85^ including higher degrees of regional Aβ plaques in DLB than PDD.^84^^,^^86–88^ Overall, we can conclude that it is impossible to distinguish these two phenotypes on an individual examination without considering the temporal sequence of the event.^62^^,^^89^^,^^90^ In this context, our findings of similar Aβ percentage in PDD/DLB subgroups, and previous PET imaging studies of similar uptake in PDD and DLB using different tracers (PIB/F18-fluorbetapir/FBB),^91–93^ support only a marginal contribution from Aβ in differentiating LBDs cognitive progression trajectories.

We think that this study provides relevant evidence in establishing the role of amyloid in Lewy Body Disorders. For the first time, thanks to the use of concomitant PET/MRI scanning we extracted and compared valuable structural and molecular data.

Second, we covered the whole spectrum of Lewy Body Disorders and included a prospective evaluation at 1 year to assess cognitive worsening.

Finally, our LBDs-Aβ+ group did not differ in age, gender, clinical and behavioural features from LBDs-Aβ− patients. Specifically, our LBDs-Aβ+ group did not show differences in motor severity or motor onset, prevalence of postural instability and gait disturbance phenotype, age at symptoms onset, dopamine dose and kind as well as in neuropsychiatric functioning.

We acknowledge our study has limitations. First, only patients with early or moderate dementia were included, with no neuropathological confirmation and therefore, prospective findings should be taken with caution. Moreover, the results of similar rate of Aβ+ in PDD and DLB may be potentially biased due to the small sample size. In addition, recruitment source can also be a confounding factor as our patients were identified mainly at the movement disorder clinic rather than by the dementia centre (only 2 DLB), where patients may be more likely to have comorbid Alzheimer's disease.

Given the small sample size and the short follow time, subgroup differences should be confirmed in larger study. Increased statistical power is needed to definitively exclude amyloid deposition as a feature that distinguishes DLB from PDD. Finally, our Aβ+ patients were diagnosed based on Alzheimer's disease-range cortical Aβ deposition. Evidence suggests that lower Alzheimer's disease-range threshold may have clinical relevance in Lewy body diseases.

In conclusion, our study shows that the presence of amyloidosis plays an integral role in the neurodegenerative progression in LBDs and aggravates global cognition (MoCA, MMSE), attentive/executive and semantic recall abilities. Nevertheless, amyloidosis is not mandatory for dementia in Lewy Body Disorders as half of our demented patients were Aβ−. Moreover, the cognitive and neuroimaging profile in our LBDs-Aβ+ cohort does not present the typical morphological AD cognitive pattern since we did not observe both long-term memory decline, neither MRI AD-like atrophy pattern. Finally, the presence of Aβ does not differentiate demographic and clinical profile in Parkinson’s disease subgroups and DLB. Our findings provide grounds for an unspecific contribution of Aβ to dementia and cognitive dysfunction in Lewy body disorders.

## Supplementary material

Supplementary material is available at *Brain Communications* online.

## Supplementary Material

fcab180_Supplementary_Data

## Abbreviations

ADL =: activity of daily living
Aβ =: amyloid deposits
Aβ+ =: amyloid-positive
Aβ− =: amyloid-negative
AS =: Starkstein Apathy Scale
BAAD =: The Brain Anatomical Analysis using Diffeomorphic deformation
BDI-II =: Beck Depression Inventory-II
DEM =: Lewy body type dementia
DLB =: dementia with Lewy Bodies
FMM =: [^18^F]flutemetamol
GCA =: the Pasquier's Global Cortical Atrophy scale
GCA-F =: the Pasquier's Global Cortical Atrophy scale-frontal subscale
LBD-Aβ+ =: Lewy Body disorders with significant Aβ
LBD-Aβ− =: Lewy Body disorders without significant Aβ
MDS-UPDRS =: Movement Disorder Society Unified Parkinson’s Disease
MDS-UPDRS-III =: Motor section of Movement Disorder Society Unified Parkinson's Disease
MMSE =: Mini Mental State Examination
MoCA =: Montreal Cognitive Assessment
MTA =: medial temporal atrophy
PA =: the Koedam's scale of Posterior Atrophy
PD-Aβ+ =: Parkinson’s disease patients with significant Aβ
PD-Aβ− =: Parkinson’s disease without significant Aβ
PDD =: Parkinson’s disease dementia
PD-MCI =: Parkinson’s disease with mild cognitive impairment
PD-NC =: Parkinson’s disease with normal cognition
PDQ-8 =: Parkinson’s Disease Questionnaire
STAI Y-1 =: State-Trait Anxiety Inventory forms-state
STAI Y-2 =: State-Trait Anxiety Inventory forms-trait state
SVM =: support-vector machine
TIV =: Total Intracranial Volume
VOIs =: volume of interests
