# Supplementary material for: The contribution of beta-amyloid to dementia in Lewy body diseases: a 1-year follow-up study
Source: Brain Commun. 2021 Aug 19;3(3):fcab180. doi: 10.1093/braincomms/fcab180 (PMC8390473; doi:10.1093/braincomms/fcab180)
Supplement: fcab180_Supplementary_Data [file fcab180_supplementary_data.docx]

**Supplementary Table 1. Comparison of clinical, motor and neuropsychological characteristics (mean and SD) between Dementia (DEM) subgroups.**

|  | **DEM** | | **Mann Whitney**  **U test** |
| --- | --- | --- | --- |
|  | DLB (n = 10) | PDD (n = 13) | P value |
| *Demographical* |  |  |  |
| Age (years) | 73.11(6.37) | 73.643(3.95) | 0.8494 |
| Sex (Male) | 78% | 71% | 0.9990 |
| Education (years) | 12.20(7.36) | 9.08(6.10) | 0.3267 |
| *Clinical characteristics* |  |  |  |
| Aβ+ (%) | 40% | 54% | 0.8119 |
| Age at symptoms’ onset (years) | 69.44(7.58) | 61.50(5.51) | **0.0210^*^** |
| Disease duration (years) | 3.89(2.84) | 12.43(6.54) | **0.0007^*^** |
| LEDD (mg tot/die) | 314.72(237.41) | 665.54(307.87) | **0.0066^*^** |
| DAED (mg tot/die) | 13.33 (40.00) | 31.14(39.60) | 0.1107 |
| DA (%) | 11% | 50% | 0.0858 |
| Anticholinesterase (%) | 2/10 | 5/14 | 0.6529 |
| Hallucination (%) | 67% | 79% | 0.6430 |
| *Motor characteristics* |  |  |  |
| MDS-UPDRS-III | 33.50(10.86) | 49.43(26.80) | 0.2598 |
| PIGD phenotype (%) | 89% | 100% | 0.3913 |
| H&Y>3 (%) | 22% | 64% | 0.0894 |
| *Functional Activities* |  |  |  |
| IADL | 3.00(1.73) | 2.07(2.02) | 0.2245 |
| ADL | 4.30(1.70) | 2.62(202) | 0.0513 |
| PD-CFRS | 13.80(6.34) | 15.81(6.28) | 0.3194 |
| *Global cognitive status* |  |  |  |
| MMSE | 19.22(3.78) | 18.15(3.71) | 0.5705 |
| MoCA | 13.68(3.83) | 13.32(3.37) | 0.3259 |
| *Behavioral measures* |  |  |  |
| PDQ-8 | 8.66(7.37) | 14.50(6.53) | 0.2997 |
| BDI-II | 6.17(3.97) | 13.83(8.23) | 0.1276 |
| AS | 18.40(8.08) | 22.80(5.71) | 0.2506 |
| STAI-Y1 | 36.00(4.47) | 42.83(9.81) | 0.1275 |
| STAI-Y2 | 39.67(9.61) | 46.20(12.28) | 0.4631 |
|  |  |  |  |

*Note.* H&Y: Hoehn‐Yahr; PIGD phenotype (%): percentage of postural instability and gait disturbance; LEDD: levodopa and dopamine agonist equivalent daily doses; DAED: dopamine agonist equivalent daily dose; DA (%): percentage of patients in dopamine agonist therapy, IADL: instrumental activities of daily living; PD-CFRS: the Parkinson’s Disease Cognitive Functional Rating Scale; **^*^** P < 0.05

**Supplementary Figure 1.** **Age distribution density among cognitive subgroups**


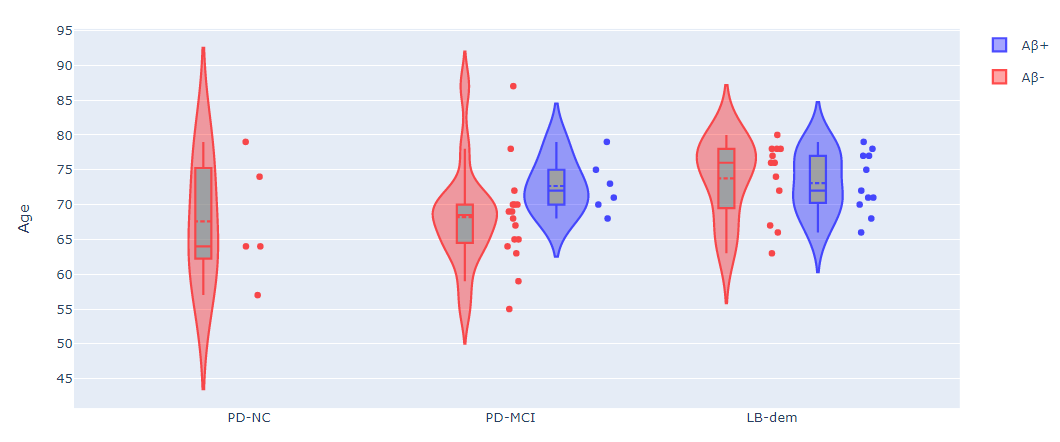


Note. Violin plot showing the age distribution among cognitive subgroups: violin width shape indicates the probability density at each age value detailed in the dot-plot.

Supplementary Table 2. **Percentage of AD-like indexes in PD-MCI and Dem subgroups according to the Aβ status.**

|  | PD-MCI | | Dem | | Chi squared |
| --- | --- | --- | --- | --- | --- |
|  | LBDs-Aβ- (n = 15) | LBDs-Aβ+ (n = 6) | LBDs-Aβ- (n = 11) | LBDs-Aβ+  (n = 10) | p-value |
|  |  |  |  |  |  |
| AD-like VR (%) | 33 | 17 | 27 | 50 | 0.53 |
| AD-like VR plus Fazekas (%) | 7 | 0 | 0 | 0 | 0.60 |
| GCA (%) | 27 | 17 | 27 | 30 | 0.94 |
| GCA-Frontal sub-score (%) | 27 | 33 | 36 | 20 | 0.85 |
| MTA score R (%) | 7 | 17 | 27 | 0 | 0.22 |
| MTA score L (%) | 13 | 16 | 18 | 0 | 0.58 |
| PA Koedam score R (%) | 20 | 17 | 18 | 40 | 0.58 |
| PA Koedam score L (%) | 13 | 17 | 18 | 30 | 0.77 |
| Fazekas (mild Vasc.) (%) | 7 | 16 | 9 | 0 | 0.64 |
| Early AD-like automated (%) | 27 | 33 | 40 | 60 | 0.40 |

*Note.* AD-like VR: frequency of an AD-like pattern according to the presence of moderate to severe combination of MRI atrophy at visual rating scales (Global cortical atrophy-Frontal subscore, Middle temporal atrophy, posterior Atrophy-Koedam-score); AD-Like VR plus Fazekas: patient with an AD-like pattern based on visual rating scale and a moderate to severe subcortical vascular alteration; Early AD-like automated: patients with an early AD-like pattern based on an automated AD-score index using a MRI segmentation approach.
